# Improving early detection initiatives: a qualitative study exploring perspectives of older people and professionals

**DOI:** 10.1186/s12877-017-0521-5

**Published:** 2017-06-23

**Authors:** Manon Lette, Annerieke Stoop, Lidwien C. Lemmens, Yvette Buist, Caroline A. Baan, Simone R. de Bruin

**Affiliations:** 1 Amsterdam Public Health research institute, Department of General Practice and Elderly Care Medicine, VU University Medical Center, Amsterdam, the Netherlands; 2 Centre for Nutrition, Prevention and Health Services, National Institute for Public Health and the Environment, Bilthoven, the Netherlands; 3 Scientific Center for Transformation in Care and Welfare (Tranzo), University of Tilburg, Tilburg, the Netherlands

**Keywords:** Early detection, Frailty, Health, Health care, Older people, Proactive elderly care, Social care, Wellbeing, Qualitative research

## Abstract

**Background:**

A wide range of initiatives on early detection and intervention have been developed to proactively identify problems related to health and wellbeing in (frail) older people, with the aim of supporting them to live independently for as long as possible. Nevertheless, it remains unclear what the best way is to design such initiatives and how older people’s needs and preferences can be best addressed. This study aimed to address this gap in the literature by exploring: 1) older people’s perspectives on health and living environment in relation to living independently at home; 2) older people’s needs and preferences in relation to initiating and receiving care and support; and 3) professionals’ views on what would be necessary to enable the alignment of early detection initiatives with older people’s own needs and preferences.

**Methods:**

In this qualitative study, we conducted semi-structured interviews with 36 older people and 19 professionals in proactive elderly care. Data were analysed using the framework analysis method.

**Results:**

From the interviews with older people important themes in relation to health and living environment emerged, such as maintaining independence, appropriate housing, social relationships, a supporting network and a sense of purpose and autonomy. Older people preferred to remain self-sufficient, and they would rather not ask for help for psychological or social problems. However, the interviews also highlighted that they were not always able or willing to anticipate future needs, which can hinder early detection or early intervention. At the same time, professionals indicated that older people tend to over-estimate their self-reliance and therefore advocated for early detection and intervention, including social and psychological issues.

**Conclusion:**

Older people have a broad range of needs in different domains of life. Discrepancies exist between older people and professionals with regard to their views on timing and scope of early detection initiatives. This study aimed to reveal starting-points for better alignment between initiatives and older people’s needs and preferences. Such starting points may support policy makers and care professionals involved in early detection initiatives to make more informed decisions.

**Electronic supplementary material:**

The online version of this article (doi:10.1186/s12877-017-0521-5) contains supplementary material, which is available to authorized users.

## Background

Due to the expected acceleration of population ageing, health systems are challenged to offer care and support to an increasing number of older people [1]. Governments are hoping to support people to live independently in their own home for as long as possible, with support from formal and informal caregivers [2, 3]. At the same time, the prevalence of frailty increases with age [4, 5], which in turn increases the risk of adverse outcomes such as disability, institutionalization and mortality [6, 7]. Traditionally, frailty has been viewed as a unidimensional concept focusing mainly on the physical problems that older people experience [8]. Recent approaches to frailty have recognized the multidimensional character of the concept, including physical, cognitive, psychological, social and environmental life domains [9–11].

Early detection of problems related to health and wellbeing and proactive delivery of care and support are employed with the aim to help (frail) older people remain independent and self-reliant for as long as possible [10, 12–14]. Various initiatives, which we refer to as ‘early detection initiatives’, have been developed to support this aim. Examples of such interventions in scientific literature include, for example, ‘preventive home visits’, ‘geriatric care management’, ‘identification of frailty in primary care’, and ‘population-based multidimensional geriatric assessment’ [15–20]. Such initiatives have been offered in different settings (e.g. general practitioner’s (GP) practice, hospital, municipality) and aim at different target groups (e.g. hospitalized older people, community-dwelling (frail) older people, older people with low socioeconomic status (SES)) [17, 18, 20–29]. Broadly speaking, early detection initiatives can be clustered into two groups: 1. Initiatives that aim to detect older people at risk of deterioration, in order to provide a preventive programme, and 2. Initiatives that aim to detect problems (and needs) related to health and wellbeing in frail older people in order to optimize (current) delivery of care and support [30].

Many of the initiatives described in literature purported to focus on different life domains. However, in practice such initiatives tended to mainly address physical health problems [30] and as such merely applied the unidimensional view on frailty. This could imply that there are unmet care needs that fall within, for instance, the psychological, social and environmental domains of life (e.g. company, daytime activities, access to information) [30, 31]. Furthermore, as more recent studies have already suggested, older people tend to value a multidimensional and person-centred approach, and would appreciate more alignment between initiatives and their needs and preferences [30–34].

In addition, evaluations of early detection initiatives are inconclusive. Some reviews have suggested that initiatives including a multidimensional perspective may reduce functional decline [16, 29, 35], hospital admissions [16, 29], nursing home admissions [16, 29, 36] or mortality [16, 35, 36], whereas other reviews did not find any evidence for such effects [15, 37, 38]. As details about program characteristics and program implementation are often unknown, a more comprehensive evaluation of why some initiatives seem more effective than others is difficult [15, 29, 36]. Moreover, previous reviews have overlooked other related outcomes, such as autonomy and self-confidence [29]. In addition, as a recent study in the Netherlands has indicated, there are many initiatives that have not yet been evaluated, which would suggest much is still unknown about the potential benefit of early detection initiatives [30].

The large variety of early detection initiatives and the inconclusive results with regard to the effectiveness of such initiatives make it difficult to establish what constitutes best practice. More insight is needed into how to best design early detection initiatives. Taking the needs and preferences of older people into account is crucial for developing sustainable an effective initiatives [39]. Exploring what matters to older people, taking into account all life domains, could provide important starting points for improving the alignment of early detection initiatives with the needs and preferences of (different groups of) older people. Our study therefore aimed to explore: 1) older people’s perspectives on health and living environment in relation to living independently at home; 2) older people’s needs and preferences in relation to initiating and receiving care and support; and 3) professionals’ views on what could improve the alignment of early detection initiatives with older people’s needs and preferences.

## Methods

### Study design

This qualitative study consisted of two stages. In stage one (August 2015 – January 2016), older people were interviewed to gain insight in their perspectives on health and living environment in relation to living independently at home, and to identify their needs and preferences for initiating and receiving care and support. In stage two (March 2016 – April 2016), professionals in proactive elderly care (i.e. an outreaching, proactive and integrated approach of older people living in the community, intended to prevent (further) decline in older people’s functioning and wellbeing and/or to support them to live in their own homes for as long as possible) were interviewed. During these interviews, professionals reflected on the results of the interviews with older people, and indicated how, according to them, early detection initiatives could be better aligned with older people’s needs and preferences in practice.

### Study sample

#### Stage one: Older people

We aimed to obtain insights from a wide variety of older people, including people from different age groups and educational levels. As we were interested in the needs of people currently aged over 65, as well as in the expectations of future older people, respondents were eligible to participate if they were 55 year or older. Additional inclusion criteria included living independently at home and being cognitively able to participate.

Participants were recruited in two ways. First, we put an invitation letter in a national magazine aimed at older people, and in the newsletter of the umbrella organisation for Dutch elderly organisations. Second, we approached several of our contact persons at community centres, adult day services centres and elderly organizations, who then approached older people to provide information about the study and ask them to participate. A total of 83 respondents signed up to participate, of which 58 respondents signed up via the first procedure and 25 respondents via the second procedure. Among these potential participants, maximum variation sampling [40] was used in order to obtain a diverse study population, aiming for variation in gender, age, living situation (alone or with partner), degree of urbanization of their place of residence and educational level. One respondent did not show up to a group interview, and seven respondents who signed up to participate refrained from participating when they were contacted to schedule an interview. Reasons for this included illness, not being interested in participating anymore and no longer seeing the value of participating.

Interviews were conducted until all researchers were confident no new data would emerge. Data saturation was reached after interviews with 36 participants. Of these 36 participants, seventeen had signed up via the newsletter or magazine and nineteen were enrolled via our contact persons. We personally called all remaining respondents to explain that we had not anticipated the large number of responses to our call, and to let them know they would not be invited for an interview. During those phone calls we responded to any questions or comments that they had.

#### Stage two: Professionals in policy and practice

A broad range of professionals in proactive elderly care was asked to participate in an interview. The sample included professionals in primary and social care, managers from care organisations, academics, community policy officers and representatives of organizations for older people. Professionals were selected 1) from the researchers’ network, and 2) from research and practice in early detection, brought to the researchers’ attention through literature and oral presentations during (scientific) conferences. Professionals received an email invitation to participate in the study. A total of thirteen professionals were approached, all of whom agreed to participate. In a few cases, professionals suggested to invite additional colleagues to join them in the interview. Ultimately, thirteen interviews were conducted with a total of nineteen professionals.

### Data collection

#### Stage one: Interviews with older people

Prior to the interviews, participants were asked to fill in a questionnaire to collect demographic information, including age, gender, educational level, marital status, living situation, degree of urbanization and cultural background.

Interviews were conducted by ML, SdB, LL and AS using a semi-structured interview guide. For the development of the interview guide, the authors built on the different domains of frailty [9–11] and as such focused on the physical, cognitive, psychological, and social domains of life (in this study summarized as “health”) as well as the environmental domain of life. The interview guide was reviewed by an advisory committee consisting of older people working as volunteer elderly advisors and several professionals in proactive elderly care. Based on their feedback, the initial interview guide was further adapted to ensure the questions were relevant and understandable. The interview guide addressed the following topics (for full interview guide, see Additional file 1):

1. Health and living environment
  - experienced problems and needs in the different domains of life
  - expected future problems and needs in the different domains of life
  - factors enhancing and inducing (potential) problems and needs in the different domains of life
2. needs and preferences for initiating and receiving care and support
  - needs and preferences with regard to informal care and support
  - needs and preferences with regard to formal care and support

Interviews took place face-to-face at people’s home and in community centres. Initially, we aimed to interview participants in group interviews, in order to allow older people to react to each other, complement each other’s statements and to stimulate discussion. Due to travel distances between participants and/or the frail condition of some participants, eventually most participants were interviewed separately. Two group interviews were held with a total of eight participants, and 24 participants were interviewed individually. In two cases (four participants), participants who had signed up as a couple were interviewed as a couple. In two cases, a partner not participating in the study was present during an individual interview. Interviews lasted 45 to 90 min, and group interviews took approximately 120 min.

#### Stage two: Interviews with professionals in policy and practice

The interview guide was developed based on the results of the interviews with older people. A draft version of this interview guide was reviewed by an advisory committee of professionals in proactive elderly care as well. The interview guide addressed the following topics (for full interview guide, see Additional file 2):

1. Reflection on the preferences, needs and views of older people as identified in the interviews with older people;
2. Suggestions and recommendations to better align early detection initiatives with older people’s preferences and needs with regard to the target group, scope, setting, approach and timing.

Ten interviews were conducted face-to-face at the professionals’ workplace and three interviews were conducted by telephone. Interviews lasted 30 to 90 min (on average 45 min).

### Data analysis

In the sample of older people, participants aged 55 to 65 years were categorized as future older people, whereas participants aged 66 to 80 years and participants aged >80 years were categorized as older people and the eldest respectively. Although the total group of participants included both future and current older people, for the purpose of readability the total group of participants will be described as ‘older people’. The educational level of participants was categorized into low (no education, elementary school and vocational training), medium (secondary education) and high (higher education and university).

All interviews were audiotaped with the interviewees’ permission and transcribed verbatim. Data-analysis was based on the framework analysis method [41–43]. The code structure, or analytical framework, was developed based on the principles of both a deductive and an inductive approach [43]. Predetermined codes, derived from the topic list for the interviews, were used for the development of the initial framework (i.e. deductive approach). After reading several interview transcripts, additional codes were added to the analytical framework (i.e. inductive approach). When no new concepts emerged from reviewing data, the analytical framework was finalized (see Additional file 3) and used to assign codes to relevant passages of the interview transcripts [42, 43]. ML coded the transcripts of the interviews with older people and YB coded the transcripts of the interviews with professionals. AS, LL, and SdB checked the coded transcripts and discussed differences in order to reach consensus. Software for qualitative data analysis (MAXQDA 11.0.9b and 12) was used to aid in the analysis of the coded transcripts by sorting data according to codes and themes. Data from the individual interviews and group interviews with older people were analysed separately. Patterns and themes in both data sources were compared to see whether they validated or contradicted each other. Whenever there was enough data, we checked for different patterns in the data between different groups of older people with regard to gender, age, education level and marital status. Emerging themes were discussed between ML, YB, LL, SdB and AS and then clustered according to the initial research questions. Draft study findings were shared with several respondents (including older people, academics, municipality policy officers and care and support professionals, *n* = 9) to validate findings through ‘member checking’ [44]. We inquired whether the results made sense to respondents from different viewpoints. They confirmed our results and provided valuable comments, helping us to further refine our findings.

### Ethics statement

The Medical Research Ethics Committees United has assessed the study proposal and concluded that the Medical Research Involving Human Subjects Act (WMO) does not apply to this study, and official approval was not required (reference number W15.042). All participants provided written informed consent.

## Results

### General characteristics of participants

Tables 1 and 2 show characteristics of the two groups of participants. Among older people, the average age was 78 years, ranging from 58 to 96 years, and one third of the participants were male. With regard to the professionals we interviewed, five were researchers, five were community policy officers, five were health or social care professionals, two were managers of care organisations and two were working as representatives of older people.

**Table 1:** Demographic characteristics of the sample of older people (*n* = 36)

		*Number*
Age	≤ 65 years	*5*
	66–80 years	*19*
	>80 years	*12*
	Mean age (range)	*78 years (58–96)*
Gender	Male	*12*
	Female	*24*
Educational level	Low	*9*
	Medium	*10*
	High	*12*
	No information	*5*
Marital status	Married	*11*
	Unmarried	*9*
	Divorced	*5*
	Widow(er)	*11*
Living situation	Alone	*11*
	With partner	*24*
	With children >18	*1*
Degree of urbanization	City	*27*
	Village	*9*
Cultural background	Dutch	*32*
	Other	*4*

**Table 2:** Demographic characteristics of the sample of professionals (*n* = 19)

		*Number*
Occupation	Academic	*5*
	Community policy officer	*5*
	Health or social care professional	*5*
	Manager of care organisation	*2*
	Representative of association for older people	*2*
Gender	Male	*4*
	Female	*15*

Our findings are presented in three sections, corresponding to the three aims of our study. The first two sections are primarily based on data from the interviews with older people. Where appropriate, the professionals’ reflections are included in these sections as well. The results in section three are based solely on the interviews with professionals. Throughout the results, quotes are presented to illustrate our findings. To reflect the diversity of our sample, quotes were selected from older people of different sexes, age groups, educational levels, marital status and living situations. Also when selecting quotes of the professionals, we took differences in their backgrounds (e.g. health care, social care) into account.

### What are older people’s perspectives on health and living environment in relation to living independently at home?

The interviews with older people confirmed that aging at home is important to older people, and most of them explicitly indicated they want to live independently at home for as long as possible. This section provides insight in older people’s perspectives on living independently at home and what matters to them in the different domains of life. Important themes emerging from the interviews were related to different life domains: mobility, cognitive functioning, sense of purpose and autonomy, social relationships and appropriate housing and living arrangements. Each theme is discussed further below. Although they are discussed separately, it should be noted that from older people’s perspective, all life domains were interrelated and considered of importance in order to be able to live independently at home. They can therefore not be seen in isolation, as problems in different domains are often related to each other.

#### Mobility

Most of the older people we interviewed suffered from (chronic) health problems. However, with medical treatments and clever tricks, they indicated that they felt able to deal with the consequences of their health problems. The interviews highlighted that the implication of physical problems on their mobility was an important issue. With decreasing mobility, older people discussed their fears of becoming homebound and more dependent on others. This could have a negative impact on their confidence regarding their ability to live independently, as well as on other important aspects of life such as their sense of purpose and autonomy, their social life and the appropriateness of their home environment (see themes below). As one of the participants stated:

*That’s what it all depends on. The moment my ability to walk decreases, it’s over.*

*(Older participant 1, age group >80)*

Although remaining mobile was important for older people in all age groups, they talked about it in different ways. For the youngest two age groups, remaining mobile was perceived as being able to go places independently (e.g. visit friends and family, partake in activities), whereas in the oldest age group it was perceived as being able to manage at home without help (e.g. move around the house, do their own groceries shopping).

#### Cognitive functioning

Although older people were asked about (potential) problems with cognitive functioning, this domain was not extensively discussed. They often appeared not to be able or willing to discuss the subject any further. Other participants indicated that cognitive functioning was essential for their ability to live independently and some of them feared potential cognitive decline. As with mobility, also cognitive decline was associated with consequences on other domains, such as loss of autonomy and social relationships.

*I think when your mind deteriorates you’ll be in trouble, and I hope I won’t have to experience that.*

*(Older participant 2, age group 66-80)*

#### Sense of purpose and autonomy

Older people outlined that ageing healthily also encompasses living a meaningful life and having a sense of purpose. They indicated that they want to feel useful and undertake meaningful activities. However, issues like fatigue, immobility and dependency on others can be barriers. In the two oldest age groups, people indicated they feel like they do not matter anymore and are no longer of interest to others. They did not always feel useful or respected. As someone said:

*Well, it’s just that people, you’re not interesting anymore […] I mean, it’s probably just the feeling one gets, it’s probably not the reality, but the feeling that you’re no longer of interest to anyone anymore.*

*(Older participant 3, age group >80)*

*Feeling like you’re part of it [society], but also being treated with respect. Because we older people shouldn’t get the idea that we don’t matter anymore and that we’re nothing but a nuisance. And that’s the way many elderly feel right now.*

*(Older participant 4, age group 66-80)*

Older people also spoke about the importance of being in control of their life, and mentioned their wish to decide for themselves on how to arrange their different aspects of their life, such as the care and support they receive. They (expect to) experience a loss of control over their life upon becoming dependent on others (e.g. due to physical or cognitive disabilities). Older people suggested that their sense of control would be improved by being allowed and enabled to do the things they are still able to do.

*As long as this [points to head] is still good, I still can, I’m still able to decide for myself what happens to me and have the freedom to do so.*

*(Older participant 4, age group 66-80)*

#### Social relationships

Older people often mentioned the importance of having social relationships and a social network. However, the amount of time and energy people are willing or able to invest in relationships varied. Older people who indicated to be content with their social network highlighted the importance of taking initiative and remaining interested in society. They were often actively participating in the community by doing volunteer work, joining clubs and societies, and visiting local neighbourhood facilities. As one participant said:

*And third, of course, is maintaining your social contacts. It’s very important to actively invest in those and not to sit around waiting for people to come to you.*

*(Older participant 5, age group 66-80)*

Older people who were less content with their social network indicated that they experienced various barriers to investing in and maintaining social relationships, including being homebound due to disabilities or immobility, friends and family passing away, lack of financial means to partake in activities and the feeling like others are no longer interested in or available to them. Older people did not mention loneliness per se; rather, they mentioned a decrease in social contacts by describing issues such as: “I don’t get a lot of visits”, “my children live far away”, “my children are very busy” or “I don’t get out much anymore”. Interview data showed a pattern that may indicate differences in educational level between the two groups of participants, with the group of participants who were content with their social network consisting mostly of higher educated people, and the group of participants who were less content consisting mainly of lower educated people.

#### Appropriate housing and living arrangements

With regard to being able to live independently, several issues emerged from the interviews. First, older people stressed the importance of living in close proximity to facilities such as the grocery store, public transportation and the pharmacy. In addition, although older people indicated to prefer to age in a home environment, they were not unanimous as to where they preferred this to be. The older people we interviewed could be roughly divided into three categories: those who wanted to remain in their current (sometimes senior-unfriendly) home, those who preferred to move to a more senior-friendly home and those who had already moved to a more senior-friendly home. Most of the participants in the last category indicated to have decided to move after their partner passed away. Several participants who preferred to move to a more senior-friendly home were not (yet) able to, for instance due to financial constraints or a lack of appropriate houses in the area. Other older people indicated that they would like to age in their current home and remain in their own neighbourhood where they were already familiar with the people living there and the available facilities. As someone said:

*Well see, the comfort of having lived somewhere for a long time, such as I have now, is knowing people very well. Thus [I am aware that] they know that when I need help with something, it’s okay for me to come to their door and ask: ‘Would you mind helping me for a minute or would you mind doing this for me?’*

*(Older participant 6, age group >80)*

Older people who did not yet live in appropriate and senior-friendly housing indicated that they need, or would need in the future, some home adaptations (e.g. stair lift, grips in the bathroom) to make their home more senior-friendly. High costs of such adaptations were mentioned as a source of concern for some of these people.

The interviewed professionals stressed the importance of remaining in one’s own community, although they indicated the availability of affordable senior-friendly houses is often a problem. More generally, they recognized that the financial costs of ageing in their own environments can be problematic for older people.

### What are older people’s needs and preferences in relation to initiating and receiving care and support?

This section focuses on older people’s views with regard to care and support and how, in their opinion, problems and needs should be addressed in order for them to be able to feel healthy and live independently for as long as possible. Themes that emerged from the interviews were: own responsibility and asking for help, formal and informal support network, anticipation of future needs and information supply about care and support services.

#### Own responsibility and asking for help

Older people often indicated to feel confident in their ability to fulfil their current needs themselves. They indicated the need to invest in staying healthy by maintaining a healthy lifestyle and preventing mobility losses, for instance by eating healthy, exercising, taking medication and using support devices (e.g. walking stick, walking frame) to prevent falling. In addition, they felt it is their own responsibility to resolve issues in the psychological and social domains of health and they prefer not to ask help for these types of problems. As someone said when asked about whether she would ask support for psychosocial issues:

*No, that’s your own responsibility. You’d need to pull yourself up by the bootstraps.*

*(Older participant 7, age group >80)*

Again, older people found it important to feel in control with regard to the care and support they receive. Participants indicated that if they would need some help, they would prefer to ask for it themselves, instead of having help and support imposed upon them. In addition, older people indicated to prefer to ask help from someone they know and whom they trust. They also stressed the importance of being listened to, being taken seriously and not being overruled by care professionals.

*So she’d make a plan based on what she thinks is necessary, but she won’t overrule me, she always tells me what she’s going to do and why, and I like that. That way, you’re respected as a person and that’s very important to me.*

*(Older participant 8, age group >80)*

*It’s just, if you already know someone, with whom you already have some sort of connection, it’s easier to ask for help from that person, than from someone you don’t know at all.*

*(Older participant 9, age group 66-80)*

The professionals we interviewed indicated that in their experience, older people are often reluctant to ask for help. With this in mind, they recognized the importance of trust and continuity with regard to care and support.

#### Formal and informal support network

Older people indicated that in order to be able to live independently, they (expected to) need practical support in carrying out (instrumental) activities of daily living. For example, participants often indicated to need domestic help, and they also indicated that help with bathing, clothing, grocery shopping, home and garden maintenance, transportation and administration were needed or would be needed in the future. Help to fulfil such types of needs may be offered by both the formal and informal support network. The interviews revealed that for needs related to (instrumental) activities of daily living, older people preferred to receive help from the formal support network. Some participants would ask organized volunteers to help with home and garden maintenance. The informal support network was preferably only used incidentally (e.g. transport to hospital). Participants indicated that they would not feel comfortable receiving structural care from their informal support network or that they do not have an informal support network that could provide this kind of care. As one woman put it:

*Well, you see, help from family and friends, it’s just, the government is suddenly completely focusing on that. It used to be natural and in many of our cultures it still goes without saying, but I think the problem is, at least here in the Netherlands, I barely ever see my neighbours. Do you think I would ask them if I ever needed something? No, I wouldn’t do that.*

*(Older participant 10, age group 55-65)*

Professionals indicated that having a support network close-by (i.e. in their neighbourhood/community) is very important for older people, both for social contacts and practical help. Some professionals stated in this context that they would like to see that people in the community looked after each other more often.

#### Anticipating future needs

Older people often believed they managed fine at the moment. When asked if they ever worried about the future, almost all older people indicated they don’t, as they ‘live by the day’. They felt it was useless to worry about what the future would bring, because they believed it to be impossible to prepare for all possible outcomes. As one participant mentioned:

*It’s not that I’m evading the responsibility, it’s just that I think it’s a waste to worry about things you won’t be able to do anything about anyway, not right now at least. […] Yeah, so I’m not really thinking about potential solutions I might someday need, because I would have to prepare for the entire repertoire and that would be a waste of energy.*

*(Older participant 8, age group >80)*

Although the older people we interviewed actually did indicate that they expected some problems in the future (e.g. mobility loss, cognitive decline, home adaptations, as indicated in part one of the results section), this did not necessarily mean they were also looking for potential solutions to these problems. As described above, people preferred to deal with their problems in their own way and in their own time.

In contrast, professionals indicated that older people often tend to overestimate their self-reliance. Therefore, they stressed the importance of early awareness among older people, indicating that instead of offering solutions to problems that could have been addressed at an earlier stage, they would rather provide older people with tools to anticipate and prevent these problems. As one professional put it:

*You know, it’s just remarkable how often I hear older people say that everything is fine. For instance this blind woman, 92 years old and with several disabilities, she says: “Well, I still do my own grocery shopping, but not when it’s slippery during winter, I don’t dare to risk it.” Well, you wonder, who does it for her when it’s slippery? A friend of hers, who’s also 90. And she says: “Yes, everything is fine, I don’t need anything, all is going well.” And when I asked: “What if something happens to your friend, what then?” Well, it seemed she’s never even thought about that.*

*(Professional 1, social care)*

#### Information about care and support services

Results from the interviews show that older people get information about health, wellbeing, housing, care and support in many ways. Some older people highlighted they used the internet, while others indicated to prefer information on paper, for instance from the municipality, health and social care professionals, community centres and elderly associations. In addition, older people mentioned they also remain informed via peers, their children, radio and television. Older people also indicated that they would go to their GP if they had any questions about either health or social problems, expecting their doctor to refer them to the right place. As one participant said:

*Well, just to my general practitioner. I always go to my GP and I’m sure he would refer me to the right place.*

*(Older participant 11, age group 66-80)*

Different patterns in information seeking behaviours were observed between lower and higher educated older people. Whereas higher educated older people often actively searched for information themselves, lower educated older people often indicated to receive their information through pamphlets in their mail and via their social network.

### What is necessary to improve alignment of early detection initiatives with the needs and preferences of older people?

Professionals were asked to share their views on the steps necessary to better align early detection initiatives with the preferences and needs of older people, taking the outcomes of the interviews with older people as a starting-point. This section describes the themes that address the most notable improvement areas for initiatives: target groups, scope, setting, approach and timing.

#### Target groups

Professionals recognized the themes mentioned by older people participating in this study and indicated that these issues are indeed relevant for many older people they see in their daily work. In addition, professionals indicated that some groups of older people are insufficiently reached by current initiatives, including older people with a low socioeconomic status, older migrants, older people with psychiatric problems, care avoiders and socially isolated older people. According to professionals, early detection initiatives should be targeted at these specific groups rather than on older people in general, taking into account specific issues that are often prevalent in these groups, such as illiteracy, limited financial means or cultural differences. As someone said:

*For example, a mosque is a place where you might inform older people and say: ‘This is the help and support you could ask for. It would be great if your children would arrange that, but if that’s not possible you may ask for help there’. It also creates awareness among those people, like ‘you’re entitled to that too’, that they’re aware of it and indicate when they need it.*

*(Professional 2, manager of care organization)*

#### Scope

Although older people appeared to regard issues relating to the psychological and social domains as their own responsibility, professionals stressed that early detection initiatives should pay more attention to problems regarding a lack of purpose, meaningful activities and social relationships. In their opinion, older people should be enabled to participate in society, particularly when they themselves experience barriers in doing so. As a professional stated:

*The loneliness and [lack of] engagement. That you’re part of society. I think that’s very important to people. And you’re only able to participate in society if you’re mobile, mentally healthy and if you have a social network.*

*(Professional 3, health care)*

#### Setting

Early detection initiatives are set within a variety of care settings, such as GP practices, municipalities or hospitals. Professionals suggested that early detection initiatives would be better situated within the community care setting, in the older person’s neighbourhood. According to them, it is important that detection of problems and risks takes place in settings where older people feel comfortable, for instance in their own homes, at their GP practice or at facilities they visit regularly. For this to be successful, communication and alignment between the different care and support professionals in a community, as well as volunteers and informal caregivers involved, is essential and may be stimulated by for instance a community liaison professional. Community liaisons can provide a bridge between community and the health and care professionals, making sure signals are picked up by the appropriate people.

*So one centre where health and social care are close together, and where citizens in particular can do all sorts of things, such as exercising, reading, dancing, eating, cooking, we can include nutrition of course. […] And it could also have a signalling function, as volunteers who work there will notice that you didn’t come in today or that you’ve become confused or that your appearance has changed. Such a centre shouldn’t be very complicated to set up but could be very beneficial for older people.*

*(Professional 4, health care)*

#### Approach

When talking about what would be the best approach to early detection, professionals felt that a one-size-fits-all approach, not taking into account older people’s diverse range of needs and preferences, is a potential hazard. Professionals advised a more person-centred approach is important for aligning early detection initiatives with older people’s needs, for instance by training care providers in motivational interviewing and providing older people with relevant information about their options for care and support. Information should be tailored to specific target groups and provided in a way that matches people’s information seeking behaviour.

*From our perspective, where we see opportunities for improvement, I think, is to differentiate and align to their needs. Information provision, we also have a role in that, and in applying, in improving the integration in our work. And, above all, working together with the elderly.*

*(Professional 5, community policy officer)*

#### Timing

Since, according to professionals, older people do not always appear to be open to early detection initiatives and may perceive them as patronizing, they indicated that initiatives should be timed more specifically. Older people might be more receptive of early detection initiatives when a potential shift in needs occurs. For example, needs of older people may change after certain life events, such as moving house, becoming widowed or after hospitalization. Other appropriate moments to introduce early detection initiatives could include, for example, when signs of cognitive decline or fall incidents occur, or in case of changes in laws and regulations concerning care and support. In addition, some professionals advocated the need to raise awareness of (prevention of) potential problems and risks at a relatively young age, for instance when people retire from work. As one professional explained:

*Well, then you’ll see that the more frail someone becomes, the less capable this person will be to proactively go out and about in their neighbourhood and expand their social network. That’s why, in our mission, we’ve been focusing on the people retiring from work who don’t have any health complaints yet. They are fit and healthy and are…, should be able to invest in expanding their network. Those people should be stimulated to do just that, before they become too frail to actively invest in their social network.*

*(Professional 6, social care)*

## Discussion

Over the years, in several countries initiatives have been implemented to proactively identify problems related to health and wellbeing in frail older people [15, 16, 29, 30, 35–38]. Despite a wide array of studies, it remains unclear how such initiatives can be best designed and implemented to address the needs and preferences of older people. In this study, we explored older people’s perspectives on health and living environment in relation to independent living at home, their needs and preferences in relation to initiating and receiving care and support, and professionals’ views on what might be necessary to improve the alignment of early detection initiatives with older people’s needs and preferences. Our results show that important prerequisites for living independently at home are maintaining mobility, healthy cognitive functioning, appropriate housing and a supporting network. In addition, maintaining independence, social relationships, a sense of purpose and autonomy are important to age in a meaningful and respectable manner. This study also highlights several discrepancies between perspectives of older people and the perspectives of professionals. Awareness of such discrepancies and their implications for policy and practice is important, and a dialogue between both parties, as well as other relevant stakeholders, is necessary to further explore such contradistinctions.

As also suggested by earlier studies [31, 45–47], this study highlights that for older people, health and independent living encompasses various health (including physical, cognitive, psychological and social) and environmental domains. Although older people often have few unmet needs concerning physical health [31], our findings regarding the importance of mobility for maintaining independence and healthy ageing are supported by previous studies. Preventing and tackling mobility loss is an important part of holistically addressing older people’s problems in various life domains, as mobility deficits are associated with health problems, a decrease in social contacts and a decreased ability to take part in society [48, 49].

In contrast to the physical, psychological, social and environmental domains of life, which were discussed in detail during the interviews, the majority of older people in our study did not elaborate extensively on cognitive health and functioning. Possible explanations for this are that cognitive decline did not play a role, or played only a minor role, in the older participants’ lives, that they felt that cognitive decline was beyond their control and they were unsure what to say about it, or because for some it is still a taboo subject [50, 51]. In addition, previous research showed that denial and concealment of cognitive problems or unawareness of cognitive decline regularly occur among people with early-stage dementia [51, 52]. Nonetheless, awareness and early diagnosis of cognitive decline may positively affect older people’s autonomy, as it enables them to be involved in the planning of their future care [53].

Autonomy is a recurring theme in the results of this study. As was also found in a previous qualitative study on older people’s views of healthy aging [45], older people want to be self-sufficient, feel in control over their life and prefer not to ask for help. When they do ask for help, familiarity and reciprocal trust are important in the relationship between the older person and his or her caregiver, as well as being respected and listened to [33, 45, 54]. The current study indicates that this feeling is not exclusive to relationships with care professionals, but is also important in other social relationships.

Enabling people to maintain social relationships is important, as loneliness and social isolation are risk factors for many negative health outcomes [55–57]. Factors found to increase the risk of social isolation and loneliness are losing a partner, decreased social activities, physical or cognitive disabilities and increased feelings of low mood and uselessness [56, 57]. As the present study shows, these are indeed prevalent issues among older people, and addressing them may be a starting point for addressing loneliness and social isolation.

The importance of the environmental domains of life is supported by recent studies as well. Having a home appropriate for their needs and being able to get around in the community are important aspects of older people’s quality of life [46, 58]. Physical and social neighbourhood characteristics, such as safety, accessibility of facilities and social cohesion and the availability of social support, may be crucial in enabling people to age in their own preferred environments [59]. As such, the availability of appropriate homes in supportive neighbourhoods is becoming increasingly important as people age.

### Implications for early detection initiatives

This study reveals some discrepancies and issues with regard to timing, scope and approach of initiatives, which are considered to be important to address when developing and implementing early detection initiatives. This and previous research revealed that older people often perceive initiatives as patronizing [30]. Older people prefer to be autonomous and self-sufficient, and whereas they highlighted being invested in staying healthy within the present moment, they are often not willing or able to anticipate or act on (potential) future problems. However, professionals believe that older people may over-estimate themselves and are often reluctant to ask for help, and that many problems could be prevented if targeted at an earlier stage. This difference in perspectives on timing will not be easily resolved. The discrepancy might be reduced when early detection is initiated around certain life events, such as losing a partner, moving house or hospitalization. As these events often increase the risk of frailty and the need for care and support [60], older people may be more receptive of early detection initiatives at such moments [61]. Additionally, signals of potential deterioration, received from either older people themselves or someone close to them, may indicate an opening for professionals to discuss possibilities for care and support with older people. To make sure these signals end up with the right professional, an integrated and coordinated care and support system is needed. More alignment between professionals from different domains is considered necessary to prevent older people from being confronted with a multitude of interventions [30].

With regard to scope and approach, a more person-centred and comprehensive approach is advised. First, we recommend focussing more on sustaining older people’s capabilities and improving independent functioning in relation to activities that are of importance to them, despite potential limitations. As also suggested in previous research [45], aging often involves accepting and adapting to limitations on various health and environmental domains. A more explicit focus of early detection initiatives on stimulating or enhancing the ability to adapt and self-manage is in line with emerging concepts such as positive health [62], reablement [63, 64] and function focused care [65], in which the ability to live a meaningful life is central. As a supportive environment could reinforce this ability [46, 58], there is a call for action to a varied group of stakeholders (including municipalities, project developers and housing corporations) to support the availability of appropriate homes in older people’s communities. Second, as our results suggest that people value autonomy and feeling in control, we further recommend that decisions with regard to care and support are made in dialogue with older people and/or their informal caregivers, and are based on the older person’s priorities [66]. For such approaches to be successful, reciprocal trust between the care professional and the older person is important. In addition, care and support professionals need to possess advanced communication skills and techniques to support deliberation and decision-making (e.g. listening, motivational interviewing) [67]. In addition, transparent and comprehensive information about the available options for care and support should be available and accessible for different groups of older people, as this will be necessary for informed decision-making [67] and will improve older people’s experience of the care and support they receive [58].

### Methodological considerations

A strength of this study is the variety of participants included. As we interviewed both older people and professionals in proactive elderly care, we were able to reveal discrepancies between ‘supply and demand’. The wide range of professionals, representing different settings of proactive elderly care (e.g. primary care, hospitals, social care and support, elderly organizations, researchers and municipalities), resulted in a broad perspective on prevalent issues among early detection initiatives from different settings.

Although we aimed to include people who differed in age, gender, urban or rural neighbourhoods, marital status and educational level, some groups (e.g. women, people living alone, older people in the 65–80 age group) are overrepresented in our sample. Furthermore, many people in our sample signed themselves up after seeing our invitation letter. This may have caused some bias in selection, resulting in an overrepresentation of less frail and more proactive older people. Therefore, our sample of older people may not be entirely representative of the total population of older people, and may not wholly correspond with the older people addressed during interviews with health professionals. However, by approaching people through contact persons at community and adult day services centres, we actively tried to include participants who were perceived to be more frail. Interviews with these participants did not yield any new results and after 36 interviews, data saturation was reached. In addition, the professionals we interviewed, of whom many encounter varying populations of older people in their daily practice, found our findings representative. This suggests our results are valid for a larger group of older people. Furthermore, after data analysis we used member checking [44] to inquire whether our results were faithfully interpreted, whether they contained errors and whether they made sense to older people and professionals.

As we used qualitative methods, this study provided insight into themes and concepts that matter to older people, and indications for differences between different groups of older people. However, based on these results we were not able to establish which issues occur more frequently than others or to compare different groups of older people to detect potential statistical differences. To answer these types of questions, future research which includes quantitative methods is recommended. Furthermore, during our interviews with older people we did not specifically focus on their experiences of early detection initiatives. Although some older people mentioned they had taken part in one or more types of initiatives, we did not investigate their experiences with specific interventions in detail, as this did not fall within the scope of this study. It should, however, be noted that a retrospective study among recent users of early detection initiatives may provide valuable insights in experiences with specific initiatives.

## Conclusions

This study explored the perspectives of older people and professionals on health, independent living and early detection of problems and needs. Older people have a broad range of needs in different domains of life. Several discrepancies exist between older people and professionals concerning their views on timing and scope of early detection initiatives. Although these issues seem to be difficult to solve, this study reveals starting-points for better alignment between interventions and needs and preferences of older people, which may support policy makers and care professionals involved in early detection to make more informed decisions.

## Additional files

Additional file 1: Topic Guide - Interviews with Older People, Detailed topic guide which was used for the interviews with older people. (PDF 268 kb)

Additional file 2: Topic Guide - Interviews with Professionals, Detailed topic guide which was used for the interviews with professionals. (PDF 238 kb)

Additional file 3: Analysis Framework, Overview of the framework used to analyse interview data. (PDF 233 kb)

## Abbreviations

GP: General Practitioner
WMO: Medical Research Involving Human Subjects Act [in Dutch: Wet medisch-wetenschappelijkonderzoek met mensen]
