# Supplementary material for: Improving early detection initiatives: a qualitative study exploring perspectives of older people and professionals
Source: BMC Geriatr. 2017 Jun 23;17:132. doi: 10.1186/s12877-017-0521-5 (PMC5482941; doi:10.1186/s12877-017-0521-5)
Supplement: Supplementary file 1 — Topic Guide - Interviews with Older People, Detailed topic guide which was used for the interviews with older people. (PDF 268 kb) [file 12877_2017_521_MOESM1_ESM.pdf]

Topic Guide - Interviews with Older People

Note from the authors: when developing this interviews guide, we built on the different domains of frailty (physical, cognitive, psychological, social and environmental). After review by the advisory board that was set up for this project, we were advised that it would be more meaningful for older people to ask about health, wellbeing and living environment, rather than about each of these domains specifically.

Problems and needs with regard to health, wellbeing and living environment

1. Currently, you are living independently at home. How is that going? What is going well, and what could be better?
2. Do you ever worry that you may become more dependent on others? What do you worry about?
3. What are your needs with regard to your health? What do you need, or expect to need, to be able to remain physically healthy?
4. What are your needs with regard to your wellbeing? What do you need, or expect to need, to be able to keep doing what matters to you and feel well?
5. What are your needs with regard to your living environment? What do you need, or expect to need, to be able to remain living independently at home?
6. If needs are already present, when and why did they emerge?
7. If needs are present, to what extent have they already been fulfilled? How?

Needs and preferences with regard to informal care and support

8. What are you doing yourself to remain healthy?
    a. Are there any things your spouse, family or friends (can) help you with?
    b. Are you aware of any possibilities to support you and your spouse/family/friends to look after your health?
    c. Do you have sufficient access to information about how to look after your health?
9. What are you doing yourself to feel well and maintain your ability to do what matters to you?
    a. Are there any things your spouse, family or friends (can) help you with?
    b. Are you aware of any possibilities to support you and your spouse/family/friends to help you feel well and keep being able to do what matters to you?
    c. Do you have sufficient access to information about how to keep feeling well and being able to do what matters to you?
10. What are you doing yourself to remain living independently in your home?
    a. Are there any things your spouse, family or friends (can) help you with?
    b. Are you aware of any possibilities to support you to remain living independently in your home?
    c. Do you have sufficient access to information about how to remain living independently at home?

Needs and preferences with regard to formal care and support

11. For which health-related problems and needs would you need help from someone other than your spouse/family/friends?
    a. From which health care worker, social worker or volunteer would you prefer to receive that help?
    b. How would you prefer they helped you?
12. For which problems and needs related to your wellbeing would you need help from someone other than your spouse/family/friends?

   a. From which health care worker, social worker or volunteer would you prefer to receive that help?
   b. How would you prefer they helped you?
13. For which problems and needs related to your living environment would you need help from someone other than your spouse/family/friends?
    a. From which health care worker, social worker or volunteer would you prefer to receive that help?
    b. How would you prefer they helped you?
14. How do health care workers, social workers or volunteers find out about your needs? How do they find out about what matters to you?

Note to interviewer: following the flow of the conversation, topics may not be addressed in this exact order. However, make sure that

1) *all of the domains above (health, wellbeing and living environment) are addressed, and*
2) *for each domain, all relevant questions (current/expected problems and needs, help from informal care network, information access and help from formal carers) are addressed.*

<u>Closing</u>

15. We talked about many things today. If you think back to all the topics we discussed, which would be most important to you to be able to live independently at home for as long as possible? Please pick three.
