# Supplementary material for: Improving early detection initiatives: a qualitative study exploring perspectives of older people and professionals
Source: BMC Geriatr. 2017 Jun 23;17:132. doi: 10.1186/s12877-017-0521-5 (PMC5482941; doi:10.1186/s12877-017-0521-5)
Supplement: Supplementary file 2 — Topic Guide - Interviews with Professionals, Detailed topic guide which was used for the interviews with professionals. (PDF 238 kb) [file 12877_2017_521_MOESM2_ESM.pdf]

Topic Guide - Interviews with Professionals

1. During the interviews with older people, several perceived and/or expected problems and risks emerged (see textbox 1). Do you recognize these issues?
    a. Which issues do you recognize?
    b. Which issues don you not recognize?
    c. Do you miss any issues based on your experience?

Textbox 1: Problems and risks emerging from interviews with older people

Physical domain
- ✓ Decrease in/loss of mobility
- ✓ (Chronic) health problems
- ✓ Pain
- ✓ Fatigue
- ✓ (Falling) Accidents

Cognitive domain
- ✓ Problems with memory

Psychological
- ✓ Sadness
- ✓ Anxiety due to disabilities
- ✓ Feeling useless and not of interest to others anymore

Social domain
- ✓ Loss of a loved one
- ✓ Limited social network:
    - o No/little contact with neighbours
    - o Children are busy or live far away
- ✓ Limited social activities:
    - o Fatigue
    - o Disabilities
    - o Insufficient financial resources?

Living environment
- ✓ Not senior-friendly
- ✓ Housebound
- ✓ Unsafety of neighbourhood and house
- ✓ Home and yard maintenance
- ✓ Difficulties with instrumental activities of daily living
- ✓ Difficulties with administration
- ✓ Insufficient financial resources for:
    - o Home adaptations
    - o Hiring help
    - o (Technological) aids

2. Based on the interviews with older people, we listed some of the most important needs in textbox 2.
    a. In your opinion, which of these needs are addressed by current early detection initiatives? Which of these needs are not yet covered? What do you think is the reason for this?
    b. Which of the unaddressed needs are, in your opinion, most urgent to address?
    c. How could, in your opinion, current early detection initiatives be improved to better align with these needs?

Textbox 2: Most important needs (based on prioritisation)

Physical domain

- ✓ Remaining mobile
- ✓ Not having any pain
- ✓ Remaining healthy and fit

Cognitive domain

- ✓ Maintaining cognitive abilities

Psychological domain

- ✓ Living a meaningful life
- ✓ Having a sense of purpose
- ✓ Being autonomous and independent

Social domain

- ✓ Having social contacts and activities
- ✓ Having a social network and social safety net

Living environment

- ✓ Remaining independently at home for as long as possible
- ✓ Being able to get out of the house / transportation facilities
- ✓ Home adaptations
- ✓ Help with home and yard maintenance
- ✓ Better information
- ✓ Safe house and neighbourhood

Based on the interviews with older people, textbox 3 describes some preferences of older people with regards to how to fulfil their needs.

Preferences with regard to fulfilment of needs

Setting

- ✓ Many older people expect their General Practitioner (GP) to know what they should do with their problems. They prefer to ask the GP, or else e.g. a community nurse or volunteer they already know and trust.
- ✓ When they have problems in the psychological or social domain (sadness, feeling unhappy, feeling lonely) older people differ in who they would ask for help.
- ✓ Many older people indicate they do not want to rely structurally on informal care. Possibly for acute relief/advice, but not long-term; they do not want to burden their children/neighbours.
- ✓ There is a high demand for senior-friendly homes.

Timing

- ✓ Many older people indicate they would ask for help themselves, if they think they need it.
- ✓ With regard to issues on the social domain, older people also indicate it is very important to take initiative.

Approach

- ✓ Older people value autonomy and respect, they want to feel like they matter. They do not want to feel like people are imposing or interfering with their lives.
- ✓ Older people want to be approached as a person, and do not like it when carers are rigidly trying use lists to make an inventory of their needs.
- ✓ Continuity in care and support staff is important to older people, they don’t want to see a new face every day.
- ✓ Periodical home visits from the GP or practice nurse are appreciated.

3. Do you recognize these preferences with regard to setting?
    a. What do these preferences, in your opinion, imply for the setting in which it is appropriate to offer early detection initiatives?
    b. How could, in your opinion, current early detection initiatives be improved to better align with these preferences regarding setting?

4. Do you recognize these preferences with regard to timing?
    a. When do you think it would be most appropriate / efficient / meaningful to offer early detection initiatives?
    b. How could, in your opinion, current early detection initiatives be improved to better align with these preferences regarding timing?

5. Do you recognize these preferences with regard to approach?
    a. Based on these preferences, which of the current approaches seem appropriate to you? Which do not?
    b. How could, in your opinion, current early detection initiatives be improved to better align with these preferences regarding approach?

6. With regard to target group, do you think that current early detection initiatives are targeting all appropriate groups? Are there groups that are not yet targeted properly?
    a. How could, in your opinion, current early detection initiatives be improved to better align with these preferences regarding target group?

7. What is your most important recommendation on how to improve early detection initiatives to better align them with the needs and preferences of older people?
    a. Who should take the lead in this?
    b. What could be your own role?
