# Supplementary material for: Improving early detection initiatives: a qualitative study exploring perspectives of older people and professionals
Source: BMC Geriatr. 2017 Jun 23;17:132. doi: 10.1186/s12877-017-0521-5 (PMC5482941; doi:10.1186/s12877-017-0521-5)
Supplement: Supplementary file 3 — Analysis Framework, Overview of the framework used to analyse interview data. (PDF 233 kb) [file 12877_2017_521_MOESM3_ESM.pdf]

Analysis framework

Main theme	Subtheme_1	Subtheme_2
Problems and needs	General	Experienced
		Expected
		(Potential) cause
	Physical domain	Experienced
		Expected
		(Potential) cause
	Cognitive domain	Experienced
		Expected
		(Potential) cause
	Psychological domain	Experienced
		Expected
		(Potential) cause
	Social domain	Experienced
		Expected
		(Potential) cause
	Living environment	Experienced
		Expected
		(Potential) cause
Things going satisfactorily	General	
	Physical domain	
	Cognitive domain	
	Psychological domain	
	Social domain	
	Living environment	
Dealing with problems and needs	General	Own initiative
		Someone else's initiative
		Initiative unknown
		Problems/needs not dealt with
		Information source
		Other
	Physical domain	Own initiative
		Someone else's initiative

**Main theme**	**Subtheme_1**	**Subtheme_2**
		Initiative unknown
		Problems/needs not dealt with
		Information source
		Other
	Cognitive domain	Own initiative
		Someone else’s initiative
		Initiative unknown
		Problems/needs not dealt with
		Information source
		Other
	Psychological domain	Own initiative
		Someone else’s initiative
		Initiative unknown
		Problems/needs not dealt with
		Information source
		Other
	Social domain	Own initiative

**Main theme**	**Subtheme_1**	**Subtheme_2**
		Someone else's initiative
		Initiative unknown
		Problems/needs not dealt with
		Information source
		Other
	Living environment	Own initiative
		Someone else's initiative
		Initiative unknown
		Problems/needs not dealt with
		Information source
		Other
Prioritisation of needs	One	
	Two	
	Three	
	Reason	
Anticipating problems and needs	Type	
	Reasons	
Experiences with early detection	Type of setting	
	Type of professional	
	Type of approach	
